# Sugar ingestion and dichotic listening: Increased perceptual capacity is more than motivation

**DOI:** 10.2478/v10053-008-0153-6

**Published:** 2014-02-20

**Authors:** Matthew H. Scheel, Aimee L. Ambrose

**Affiliations:** Department of Psychology, Carroll University, Wisconsin, USA

**Keywords:** perceptual load, selective attention, glucose, dichotic listening

## Abstract

Participants ingested a sugar drink or a sugar-free drink and then engaged in a pair of dichotic listening tasks. Tasks presented category labels then played a series of word pairs, one in the left ear and one in the right. Participants attempted to identify pairs containing a target category member. Target category words were homonyms. For example, *arms* appeared as a target in the “body parts” category. Nontargets that played along with targets were related to a category-appropriate version of the target (e.g., *sleeves*), a category-inappropriate version (e.g., *weapons*), or were unrelated to either version of the target (e.g., *plant*). Hence, an effect of nontarget type on number of targets missed was evidence that participants processed nontargets for meaning. In the divided attention task, participants monitored both ears. In the focused attention task, participants monitored the left ear. Half the participants in each group had the divided attention task before the focused attention task; the other half had the focused attention task before the divided attention task. We set task lengths to about 12 min so working on the first task would give sufficient time for metabolizing sugar from the drink before the start of the second task. Nontarget word type significantly affected targets missed in both tasks. Drink type affected performance in the divided attention task only after sufficient time for converting sugar into blood glucose. The result supports an energy model for the effect of sugar ingestion on perceptual tasks rather than a motivational model.

## Introduction

In her load theory, Lavie (1995) proposed that individuals perceive stimuli until perceptual capacity is full, regardless of relevance; and that high perceptual loads exceed perceptual capacity, thereby forcing people to focus attention. In a test of load theory, Lavie, Hirst, de Fockert, and Viding (2004) used a flanker-compatibility task that required participants to press “0” on a keypad if a *z* appeared in a search array, or press “2” if an x appeared, as quickly as possible while ignoring a flanker adjacent to the array. A low load condition featured a single target letter in the search array while a high load condition featured a target letter mixed into a randomly arranged six-letter string containing *S, K, V, J*, and *R*. A flanker was either a response-compatible (e.g., an *X* appeared when an *x* was the target) or a response-incompatible (e.g., an *X* appeared when a *z* was the target) letter that appeared either above or below the center of the search array (one experiment also included an *N* as a neutral flanker). Lavie et al. found that participants responded more slowly when an incompatible flanker appeared than when a compatible flanker appeared, but only when the search array was a single letter. Hence, flanker influence was a function of the difference between the task’s perceptual load and the participant’s perceptual capacity. Reducing the number of stimuli in the search array (i.e., *decreasing* perceptual load) increased flanker interference. Lavie et al. also found that requiring participants to perform a concurrent working memory task (i.e., *increasing* cognitive load) increased interference. Lavie et al. interpreted their results as evidence for a post-perceptual cognitive stage that actively blocks irrelevant stimuli remaining after initial perception.

### Blood glucose

A substantial body of literature shows that ingesting sugar subsequently improves performance on perceptual and cognitive tasks (Feldman & Barshi, 2007). From one perspective, glucose in the blood affects performance on cognitive tasks by acting as fuel for cognitive processes. According to this energy model, sugar ingestion should affect performance after an approximately 12-min metabolization period (Masicampo & Baumeister, 2008; McMahon & Scheel, 2010). Specifically, sugar ingestion should subsequently increase perceptual capacity, thereby reducing a task’s relative perceptual load burden.

Molden et al. (2012) recently challenged the energy model. In their study, participants rinsed their mouths with sugar-sweetened water or aspartame-sweetened water, then immediately began work on a Stroop task. Despite ingesting no sugar, and having insufficient time for metabolization, participants who rinsed with sugar-water had faster response times on the Stroop test. Molden et al. interpreted their results as evidence for a motivational model, whereby the mouth’s contact with carbohydrates motivates performance by signaling that reward is possible (for a replication, see Sanders, Shirk, Burgin, & Martin, 2012).

### Current study

Macdonald and Lavie (2011) recently extended load theory to cover auditory processing, and refined their definition of *perceptual load* to include “either the number of different task stimuli or the perceptual requirements of the task performed on the same stimuli” (p. 1780). A person who allocates fewer resources to perception should respond to a moderately demanding perceptual task as if it exerts a relatively high perceptual load. Likewise, a person who allocates more resources to perception should respond *to the same task* as if it exerts a relatively low perceptual load.

**Figure 1.**
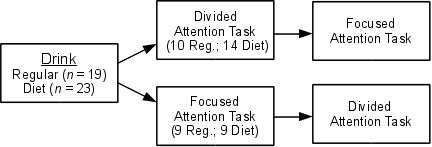
Summary of experimental design.

The current study tested if, and when, ingesting sugar affects likelihood to process words in a dichotic listening task. Participants in a *regular* group drank a caffeine-free soft drink containing sugar, and participants in a *diet *group drank a caffeine-free soft drink containing no sugar (after McMahon & Scheel, 2010). Next, half the participants in each group faced a divided attention dichotic listening task followed by a focused attention dichotic listening task; while the other half faced a focused attention dichotic listening task followed by a divided attention task (see Figure 1). We set task lengths to about 12 min so working on the first task would give sufficient time for metabolizing sugar from the drink before the start of the second task.

The dichotic listening tasks were updates of procedures published in Johnston and Wilson (1980). In the divided attention task, participants monitored both ears, and in the focused attention task, participants monitored the left ear. In our version, a computer presented category labels on a screen and then played a series of word pairs, with one word in the left ear and one in the right. Participants attempted to identify pairs containing a target category member. Our dependent variable was the number of targets missed. Target words were homonyms and nontarget words that played along with targets were related to a category-appropriate version of the target, a category-inappropriate version, or were unrelated to the target. If processed for meaning, category appropriate nontargets should facilitate target identification, whereas nontargets associated with alternate (category inappropriate) target meanings should interfere with target identification. Increased perceptual capacity should lead to an increase in ability to process both targets and nontargets for meaning. Hence, number of targets missed, as a function of nontarget type, is a measure of perceptual capacity.

Both the motivational model and the energy model predict sugar ingestion will increase the *regular* group’s perceptual capacity. However, the models differ regarding when an effect will take place. Molden et al.’s (2012) motivational model predicts an effect starting in the first task, whereas a traditional energy model predicts that an effect of sugar ingestion will emerge in the second task.

In the divided attention task, the predicted effect of sugar ingestion is relatively straightforward. Increasing perceptual capacity with sugar ingestion should improve ability to process targets and nontargets on each trial, thereby increasing the effect of nontarget type. In the focused attention task, the predicted effects of sugar ingestion are multifold. Monitoring one channel should require less perceptual capacity than monitoring two channels. The perceptual load for the focused attention task is, therefore, less than the perceptual load for the divided attention task. However, unlike the divided attention task, the focused attention task requires ignoring words in the unattended channel. According to perceptual load theory, blocking irrelevant stimuli exerts a cognitive load, rather than a perceptual load. If sugar ingestion affects cognitive capacity, then sugar ingestion should facilitate blocking irrelevant stimuli. Therefore, we predicted that nontarget type would have *less* effect on number of targets missed by the *regular* group than the *diet *group in the focused attention task.

## Method

### Participants

Forty-three undergraduates (30 females, 13 males) at a small Midwestern university volunteered. Participants ranged from 19 to 49 years old (*M* = 21.53, *SD* = 4.43). One male’s data was discarded due to an error when recording his identification number.

### Procedure

#### Taste test

Before testing, the senior researcher (M.S.) covered bottle labels with duct tape and affixed numbered stickers to bottle necks. Half were bottles of Point Premium Root Beer, sweetened with 45 g of sugar. The other half were bottles of sugar-free Point Premium Diet Root Beer, sweetened with sucralose (Point Brewery, Stevens Point, WI, USA). The experimenter (A.A.) randomly drew bottles from a refrigerator and recorded each participant’s bottle number, unaware of which type of root beer was in each bottle. This assigned participants to either a regular (*n* = 19) or diet (*n* = 23) condition. To prevent participants from hearing the experimenter opening only one bottle, A.A. prepared the taste test prior to participant arrival. She poured half the 12-ounce bottle of root beer into a disposable red cup and the other half into a disposable blue cup. Participants drank from each cup and then filled out a form indicating whether either drink was sweeter, whether one tasted better, or whether either tasted bland. We did not analyze taste test results.

#### Dichotic listening

Participants wore headphones while a computer program presented a pair of dichotic listening tasks. The program was written in Python 2.6 using the PsychoPy IDE (Peirce, 2009). Eighteen randomly assigned participants completed a focused attention task and then a divided attention task; the 24 remaining participants completed a divided attention task and then a focused attention task. In the divided attention task, the program instructed participants to attend to both ears; in the focused attention task, the program instructed participants to exclusively attend to the left ear.

Each task contained one practice block and two experimental blocks. Before each block, participants saw a target category label on the computer screen for 9 s. Focused attention task categories included child’s games, body parts, and edibles. Divided attention task categories included beverages, animals, and clothing. After category presentation, participants simultaneously heard one word in the left ear and one word in the right ear. One female voice (A.A.) read each word at conversational speed. We used a sound editing program (Audacity) to ensure each word in a pair started simultaneously, had similar durations (between 500 ms and 1,000 ms), and had the same peak volume. Each practice block included 60 word pairs, and each experimental block included 81 word pairs. Nine pairs in each block contained a target category member; the remaining 72 pairs (51 during practice blocks) contained random words.

Target category members were homonyms. Nontarget words that accompanied target words were either semantically related to a category-appropriate version of the target, semantically related to a category-inappropriate version of the target, or unrelated (neutral) to either meaning of the target (see Appendix A). The program randomly sampled word pairs without replacement. Selection of a pair containing a category member initiated a subroutine that randomly selected one of the three nontargets. In the divided attention task, targets had a 50% chance of playing on the left ear and a 50% chance of playing in the right ear.

Participants identified trials containing a target category member by pressing the right arrow key and identified trials without a target category member by pressing the left arrow key. To discourage guessing, on-screen directions instructed participants to accompany all right arrow key presses by repeating aloud the target category member they heard. The experimenter remained in the room to ensure compliance and to answer questions.

### Analyses

The dependent variable was the sum of the missed targets for each type of nontarget during experimental blocks. The program also recorded response times in milliseconds. We analyzed results from divided attention and focused attention tasks separately using 2 × 2 × 3 mixed design ANOVAs with Drink Type (regular or diet) and Task Order (focused-divided or divided-focused) as between-groups factors. Nontarget Type (appropriate, neutral, or inappropriate) was the within-groups factor. We used nonparametric tests if an ANOVA suggested potential effects (*p* < .10), but the data set failed a Shapiro-Wilk normality test. Mann-Whitney *U* tests compared across drink types, and Friedman tests with Dunn’s correction compared across distracter types.

## Results

Response time data were analyzed but were found not to be affected by drink type in either divided or focused attention tasks (all *p* values > .10) and are not reported here. The following analyses refer exclusively to the number of targets missed.

### Divided attention

An ANOVA revealed a main effect of nontarget type, *F*(2, 76) = 8.57, *MSE* = 18.10, *p* < .0001, ƞ^2^ = .171; and an interaction between Drink Type and Task Order, *F*(1, 38) = 4.18, *MSE* = 4.43, *p* = .05, ƞ^2^ = .098. However, the distribution failed the Shapiro-Wilk normality test (*W* = 0.90, *p* < .0001).

A Friedman test confirmed that nontarget type affected missed targets, χ^2^(2, *N* =42) = 15.365, *p* = .0005. A Dunn’s multiple comparison test revealed significant differences between appropriate (*M* = 1.26, sum of ranks = 66.5) and neutral (*M* = 1.83, sum of ranks = 88) nontargets (*p* < .05), and between appropriate and inappropriate (*M* = 2.57, sum of ranks = 98.5) nontargets (*p* < .001).

**Figure 2.**
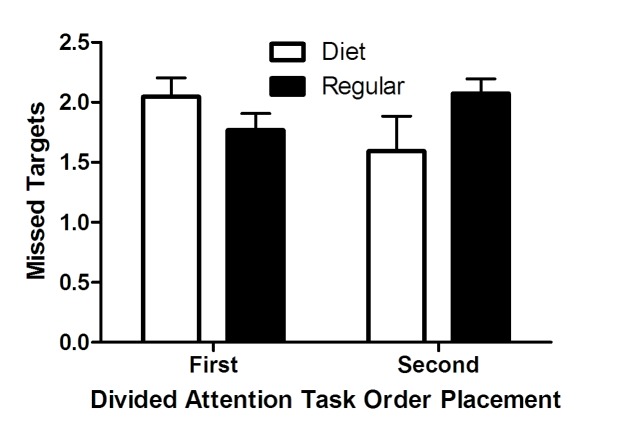
Mean targets missed during the divided attention task by drink type and task order. Error bars represent standard errors.

A pair of Mann-Whitney *U* tests clarified the interaction between Drink Type and Task Order. When the divided attention task was first, there was no significant difference in missed targets between the *regular* group (*n* = 10, *M* = 1.77, sum of ranks = 102.5) and the *diet *group (*n* = 14, *M* = 2.05, sum of ranks = 197.5), *U* = 47.5, *p* = .19. However, when the divided attention task was second, the *regular* group (*n* = 9, *M* = 2.07, sum of ranks = 109) missed more targets than the *diet *group (*n* = 9, *M* = 1.59, sum of ranks = 62.0), *U* = 17.0, *p* = .04 (see Figure 2).

### Focused attention

An ANOVA revealed main effects of nontarget type, *F*(2, 76) = 8.57, *MSE* = 6.60, *p* = .001, ƞ^2^ = .145; and task order, *F*(1, 38) = 7.55, *MSE* = 7.55, *p* = .02, ƞ^2^ = .157. The interaction between Nontarget Type and Task Order also approached significance, *F*(2, 76) = 2.89, *MSE* = 2.61, *p* = .06, ƞ^2^ = .058. The main effect of drink was not significant (regular: *M* = 1.04, *SD* = 0.98; *diet*: *M* = 1.23, *SD* = 1.18) and drink failed to interact with other factors. However, the distribution failed the Shapiro-Wilk normality test (*W* = 0.84, *p* < .0001).

A pair of Friedman tests clarified the interaction between Nontarget Type and Task Order (see Table 1). When the focused attention task was first, nontarget type had no significant effect on missed targets, χ^2^(2, *n* = 18) = 1.17, *p* = .56. However, when the focused attention task was second, nontarget type significantly affected missed targets, χ^2^(2, *n* = 24) = 13.68, *p* = .001. A Dunn’s multiple comparison test revealed that participants missed significantly fewer targets when nontargets were appropriate (*M* = 0.83, sum of ranks = 37.5) than when nontargets were inappropriate (*M* = 1.96, sum of ranks = 60.0; *p* < .01).

**Table 1.** Targets Missed in the Focused Attention Task by Task Order and Nontarget Type

		Nontarget type					
		Appropriate		Neutral		Inappropriate	
Task order	*n*	Mean	Sum of ranks	Mean	Sum of ranks	Mean	Sum of ranks
Focused-divided	18	0.89	36.5	0.56	33.0	1.11	38.5
Divided-focused	24	0.83	37.5	1.29	46.5	1.96	60.0

## Discussion

The current results support an energy model for the effect of glucose on cognition and perception. Sensing sugar in the mouth may increase motivation and affect performance. However, the interaction between Drink Type and Task Order in the divided attention task, with an effect of drink appearing when the divided attention task was the second task rather than the first, suggests that the effect of glucose as an energy source for cognition and perception is greater than the role of sugar in the mouth as a motivator.

There were significant main effects for nontarget type in both attention tasks. Nevertheless, the small number of missed targets suggests a floor-effect probably minimized the effectiveness of appropriate nontargets as facilitators of target detection and prevented normal distributions of missed-targets, which in turn necessitated using less-powerful nonparameteric statistics. A floor-effect may have also contributed to our failure to find a three-way interaction between Nontarget Type, Drink Type, and Task Order; as well as our failure to find an effect of drink type in the focused attention task. One method to test whether these results are due to a floor-effect, or whether they accurately reflect absence of effects, would be to increase the number of trials. Increasing the number of trials would increase the average number of missed targets, thereby minimizing the likelihood of encountering a floor-effect. This may explain why Johnston and Wilson (1980) used 1,296 trials, four times as many as in the current study.

Another solution for avoiding a floor-effect may be to adopt a different dependent variable, or variables. For example, response times could supplement accuracy data. Slower responding in a glucose condition, along with concurrent evidence from accuracy data, would suggest the glucose group processed more stimuli. However, glucose speeds cognitive processing (Benton, Owens, & Parker, 1994; Donohoe & Benton, 1999; Kanarek & Swinney, 1990). A glucose group could presumably process more stimuli without having a slower response time - and could even process more stimuli in less time. Therefore, failure to find an effect of drink on response time (which would have been the result in the current study), or finding faster response times in a glucose condition, would be difficult results to interpret without accuracy data.

Johnston and Wilson (1980) found that nontarget word type affected target detection in a divided attention task, but not in a focused attention task. The main effect of nontarget word type in the divided attention task of the present study agrees with Johnston and Wilson’s earlier finding. However, nontarget word type’s effect on performance when focused attention was the second task is a novel result that we did not anticipate. The anomalous result may have occurred because of carry-over from the preceding divided attention task. We inserted a practice block at the beginning of each task to help familiarize participants with the procedure during the first task, and to act as a kind of “buffer” to minimize carry-over from the first task to the second task. However, nontarget type’s greater effect on missed targets when focused attention was the second task, regardless of drink type, suggests that some carry-over across tasks probably occurred. A follow-up study could test this hypothesis by using separate groups for focused and divided attention tasks.

## Appendix A

Targets and nontargets in each category came from the Appendix of Johnston and Wilson (1980). However, over three decades have passed since Johnston and Wilson’s paper, and some once popular words have fallen into disuse. In the body parts category, we replaced *neck* with *shoulder* as a target; and used *backpack*, *travel*, and *road* as appropriate, neutral, and inappropriate nontargets, respectively. In the edibles category, we replaced the inappropriate nontarget for the target *rolls* from *Royce* to *somersault*. In the beverages category, we replaced *tab* with *coke* as a target, and changed the inappropriate nontarget from *Hunter* to *drug*. In the clothing category, we replaced *cords* with *cami* as a target; and used *top*, *barber*, and *name* as appropriate, neutral, and inappropriate nontargets, respectively.
